# Abatacept in the Treatment of Juvenile Dermatomyositis-Associated Calcifications in a 16-Year-Old Girl

**DOI:** 10.1155/2020/4073879

**Published:** 2020-05-28

**Authors:** Sukesh Sukumaran, Vini Vijayan

**Affiliations:** ^1^Division of Rheumatology, Department of Pediatrics, Valley Children's Hospital, Madera, CA, USA; ^2^Division of Infectious Diseases, Department of Pediatrics, Valley Children's Hospital, Madera, CA, USA

## Abstract

Calcinosis is a feared complication of JDM that may be seen in up to 40% of children with JDM. It is associated with negative impact on the patients' quality of life due to weakness, functional disability, joint contractures, muscle atrophy, skin ulcers, and secondary infections. Calcinosis can present as superficial nodules or plaques, larger nodular deposits extending into deeper tissue layers, accumulation of calcifications along the fascial planes of muscles or tendons, or an exoskeleton of calcium leading to limitations in mobility and joint contractures. Currently, there are no known effective treatments for calcinosis and current therapy is based on anecdotal retrospective studies and cases series. We report the case of a child with JDM-associated calcinosis with extensive intramuscular calcifications who failed conventional therapies but demonstrated improvement as evident by decrease in calcinosis and improved physical function with use of abatacept. We found that use of abatacept was associated with improvement in functional outcome and recurrence did not occur. This case suggests use of abatacept as a safe and effective treatment option for calcinosis due to JDM. Furthermore, large-scale clinical studies are needed to validate our findings and to evaluate the long-term outcomes.

## 1. Introduction

Juvenile dermatomyositis (JDM) is the most common inflammatory myopathy of childhood, with an estimated incidence of 3.2 children per million per year [1]. The disease is characterized by proximal muscle weakness and characteristic rashes. Multiple organ systems including the gastrointestinal, cardiac, and pulmonary systems can be involved and contribute to the morbidity and mortality associated with JDM. Previously, the prognosis of JDM was poor with a reported mortality of 30% [1, 2]. However, with the advent of new treatments for JDM, survival and functional outcomes have improved considerably and the reported mortality has declined and is currently estimated to be 2% [3, 4]. Despite the many progresses in the treatment of JDM, calcinosis continues to be a feared complication of the disease [5, 6].

In this report, we describe the case of a 16-year-old girl with refractory JDM and skin ulcerations as well as extensive intramuscular calcifications that were poorly responsive to conventional therapy but were subsequently successfully treated with abatacept. The purpose of this report is to share our experience to rheumatologists regarding the use of abatacept as a potential treatment option for refractory calcinosis due to JDM.

## 2. Case

A 16-year-old Caucasian female was diagnosed with juvenile dermatomyositis (JDM) at the age of 7 when she presented with moderate weakness of the proximal muscles, heliotrope rash, Gottron's papules overlying the elbows, knees, and extensor aspect of the fingers, and periungual telangiectasia. She had an elevated aldolase of 15 U/L (normal 3.4–8.6 U/L) and elevated lactate dehydrogenase (LDH) of 1100 (normal 470–750 U/L), and her creatinine kinase was 506 U/L (normal 22–198 U/L). Her aspartate aminotransferase (AST) and alanine transaminase (ALT) were 115 U/L and 110 U/L, respectively. Antinuclear antibodies, human leukocyte antigen B-27, angiotensin converting enzyme, and anti-neutrophil cytoplasmic antibodies were negative. Magnetic resonance imaging (MRI) demonstrated diffuse muscle edema and myositis. Child was started on pulse methylprednisolone (30 mg/kg/day) for three doses followed by tapering doses of oral steroids 2 mg/kg/day and methotrexate subcutaneously weekly (15 mg/m^2^). She also received monthly intravenous immunoglobulin (IVIG). She showed gradual improvement in her muscle weakness then resolution of her rash. Her disease remained relatively quiescent on hydroxychloroquine and mycophenolate mofetil.

**Figure 1:**
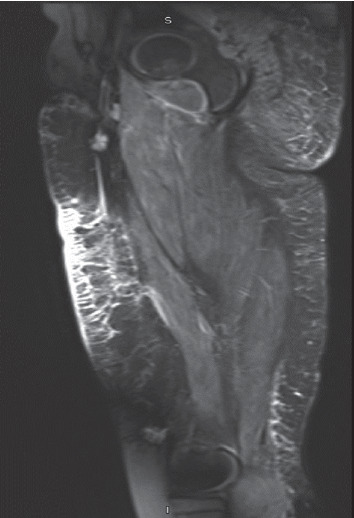
MRI imaging of thigh. Sagittal fat suppressed T2-weighted images revealing muscle edema and calcifications in the fascial planes.

At 9 years into her disease course, she presented with a 3-month history of hard masses over the buttocks and lower extremities and multiple painful, ulcerative lesions with foul smelling drainage. She denied fever, cough, difficulty swallowing, or abdominal pain but reported generalized fatigue and muscle weakness. She admitted being poorly compliant with her medications. On examination, bony hard masses ranging between 2.5 cm and 3.5 cm in diameter were palpable on her lower extremities and extended along the length of thigh to the ankle. She also had multiple ulcerative lesions over her buttocks and the posterior aspect of her thighs. Musculoskeletal examination showed symmetrical proximal muscle weakness of her pelvic and pectoral muscles, and limited range of motion of the bilateral hip, knee, and ankles. She also had lipodystrophy and muscle atrophy of her lower extremities. Laboratory investigations revealed AST 65 U/L, aldolase 12 U/L, LDH 470, and creatinine kinase 29 U/L. The rest of the physical examination were unremarkable. Her initial Childhood Myositis Assessment Scale (CMAS) score was 28 out of 52, and Childhood Health Assessment Questionnaire (CHAQ) score was 0.75. MRI of the pelvis and thighs showed extensive calcifications in her skin, in the facial planes, and muscle edema around her thighs and buttocks bilaterally. She also had muscle edema (Figure1).

Child was started on cyclophosphamide 750 mg/m^2^ in addition to high dose steroids and methotrexate. Due to the continued extension of the calcifications despite 6 months of this regimen, colchicine and probenecid were attempted with no improvement in ulcerations, calcinosis, or muscle strength. Child underwent surgical excision of the calcium deposits but continued to develop new areas of calcification and skin ulcers, and hence cyclophosphamide was discontinued. High-dose corticosteroids and IVIG were continued and, in addition, she was started on abatacept intravenously (IV) at 10 mg/kg/dose at 0, 2, and 4 weeks and then every 4 weeks. Three months after initiation of abatacept, child's cutaneous ulcerations completely healed. Her calcifications became softer and she demonstrated increased range of motion in her hip and knee joints. Her CMAS score improved to 45 out of 52, and the CHAQ score improved to 0.5 at 6 months of therapy. Her oral steroid dose was decreased by 50% at her 6-month follow-up. Nine months after starting abatacept, she was weaned off steroids completely. Plain X-rays and MRI of her lower extremities confirmed lack of progression of calcinosis, and no new lesions were detected after 1 year of treatment. She did not develop any adverse effects from abatacept.

## 3. Discussion

Calcinosis is a known contributor to the morbidity associated with JDM and can have a negative impact on the patients' quality of life, causing weakness, functional disability, joint contractures, muscle atrophy, skin ulcers, and secondary infections. Calcinosis may be seen in up to 40% of children with JDM, but the prevalence ranges anywhere from 10 to 70%. Calcinosis usually appears 1–3 years after onset of JDM but has reported to occur at onset of the disease to as long as 20 years after disease onset [6–8]. Risk factors for the development of calcinosis include a delay in the diagnosis, delay in initiating treatment or inadequate treatment, tumor necrosis factor (TNF) alpha-308A genotype, presence of autoantibodies against nuclear matrix protein 2 (NXP2), and younger age at disease onset [9, 10].

The phenotypical presentations of calcinosis are diverse. It can present as superficial nodules or plaques, larger nodular deposits extending into deeper tissue layers, accumulation of calcifications along the fascial planes of muscles or tendons, or an exoskeleton of calcium leading to limitations in mobility and joint contractures. Although calcinosis is often painless, these lesions can ulcerate and become infected. The most commonly affected sites are the buttocks, trunk, hands, feet, elbows, and knees, but lesions can develop at any site. The presentation of calcinosis differs from adults wherein calcinosis occurs in only 20% of patients and the lesions occur primarily on the extremities [6–10].

The pathogenesis of calcinosis in JDM is unknown but calcinosis also appears to occur more frequently in patients with ongoing inflammation. This finding is supported by reports indicating the presence of cells and proinflammatory cytokines such as IL-1 and TNF-alpha and a variety of proteins related to mineralization at the calcinosis site. Cytokines such as IL-6, IL-1*β*, TNF-*α*, IL-1*β*, and IL-6 were also detected in serum, suggesting the role of activated macrophages in JDM-associated calcinosis. It has also been associated with the presence of antibodies against the 140 kDa protein and with TNF-alpha-308A polymorphism [10–12].

There are no standardized recommendations for the treatment of calcinosis due to JDM, but the overarching goal of treatment is to minimize symptoms and alleviate functional limitations. Although calcium channel blockers, probenicid, and intralesional corticosteroids have been used, none of these agents have demonstrated a consistently reliable response. Many studies have reported both treatment success and failure of colchicine for calcinosis cutis. Surgical intervention can be considered in areas of noninflammatory calcinosis for treatment of pain, functional impairment, and/or skin ulceration due to calcinosis, although some reports have suggested the possibility for recurrence when underlying JDM disease activity is not well controlled [12]. Considering the high levels of TNF-alpha in patients with calcinosis, anti-TNF therapy may play a role in treating calcinosis. In 2008, Riley et al. reported a case series of five patients successfully treated with the TNF-alpha inhibitor, infliximab, for calcinosis and JDM refractory to standard therapy [13]. Given the increasing information regarding the importance of novel autoantibodies in the pathogenesis of JDM, B-cell depletion with agents such as rituximab may also prove to be effective. However, reports have suggested the possibility for recurrence when underlying JDM disease activity is not well controlled and hence control of the underlying disease process is of utmost importance.

Abatacept is a fully human soluble fusion of cytotoxic T lymphocyte antigen-4 and the Fc portion of immunoglobulin. Cytotoxic T lymphocyte antigen-4 inhibits costimulation of T-cell activation by blocking the binding of CD28 on T cells with CD80/86 on antigen-presenting cells, thereby exerting an antiinflammatory effect. Abatacept is used in the treatment of adult rheumatoid arthritis and juvenile idiopathic arthritis, but there are very limited data regarding its use for calcinosis due to juvenile dermatomyositis [14]. The mechanism of action of abatacept in JDM-associated calcinosis may be due to the inhibition of macrophages and consequent decrease in proinflammatory cytokines [14]. Arabshahi et al. reported the successful use of abatacept and sodium thiosulfate in a patient with severe recalcitrant juvenile dermatomyositis complicated by ulcerative skin disease and progressive calcinosis. This combination therapy resulted in significant reductions in muscle and skin inflammation, decreased corticosteroid dependence, and halted the progression of calcinosis. In their study, topical sodium thiosulfate was used based on its property to dissolve calcium deposits. It was not possible to determine if their patient improved due to the combination of abatacept and sodium thiosulfate or due to the sodium thiosulfate alone [15]. Our case differs in that we did not use sodium thiosulfate and hence provides further evidence for the efficacy of abatacept in calcinosis with objective radiological improvement.

In conclusion, we describe a case of refractory JDM with skin ulcerations and extensive intramuscular calcifications that were poorly responsive to conventional therapy but were subsequently successfully treated with abatacept. Our patient initially underwent surgical excision and increase in immunomodulation, but her calcinosis continued to progress which prompted use of abatacept. We found that use of abatacept was associated with improvement in her functional outcome and recurrence did not occur. As treatment late in the course of JDM has been associated with a greater amount of dystrophic calcium salt deposition, rheumatologists should explore use of abatacept early on in disease course. Further studies are needed to validate the use of abatacept for treatment of calcinosis in children with JDM and to evaluate the long-term outcomes.

## Data Availability

No data were used to support this study.

## References

[1] Mendez E. P., Lipton R., Ramsey-Goldman R. (2003). US incidence of juvenile dermatomyositis, 1995–1998: results from the national institute of arthritis and musculoskeletal and skin diseases registry. *Arthritis & Rheumatism*.

[2] Bohan A., Peter J. B. (1975). Polymyosistis and dermatomyositis. *The New England Journal of Medicine*.

[3] Huber A. M., Lang B., LeBlanc C. M. A. (2000). Medium- and long-term functional outcomes in a multicenter cohort of children with juvenile dermatomyositis. *Arthritis & Rheumatism*.

[4] Martin N., Li C. K., Wedderburn L. R. (2012). Juvenile dermatomyositis: new insights and new treatment strategies. *Therapeutic Advances in Musculoskeletal Disease*.

[5] Ramanan A. V., Feldman B. M. (2002). Clinical outcomes in juvenile dermatomyositis. *Current Opinion in Rheumatology*.

[6] Hoeltzel M. F., Oberle E. J., Robinson A. B., Agarwal A., Rider L. G. (2014). The presentation, assessment, pathogenesis, and treatment of calcinosis in juvenile dermatomyositis. *Current Rheumatology Reports*.

[7] Blance C. E., White S. J., Braunstein E. M. (1984). Patterns of calcification in childhood dermatomyositis. *American Journal of Roentgenology*.

[8] Rider L. G. (2003). Calcinosis in juvenile dermatomyositis: pathogenesis and current therapies. *Pediatric Rheumatology*.

[9] Bowyer S. L., Blane C. E., Sullivan D. B., Cassidy J. T. (1983). Childhood dermatomyositis: factors predicting functional outcome and development of dystrophic calcification. *The Journal of Pediatrics*.

[10] Pachman L. M., Liotta-Davis M. R., Hong D. K. (2000). TNF*α*-308A allele in juvenile dermatomyositis: association with increased production of tumor necrosis factor *α*, disease duration, and pathologic calcifications. *Arthritis & Rheumatism*.

[11] Mukamel M., Horev G., Mimouni M. (2001). New insight into calcinosis of juvenile dermatomyositis: a study of composition and treatment. *The Journal of Pediatrics*.

[12] Pachman L. M., Veis A., Stock S. (2006). Composition of calcifications in children with juvenile dermatomyositis: association with chronic cutaneous inflammation. *Arthritis & Rheumatism*.

[13] Riley P., Mccann L. J., Maillard S. M., Woo P., Murray K. J., Pilkington C. A. (2008). Effectiveness of infliximab in the treatment of refractory juvenile dermatomyositis with calcinosis. *Rheumatology*.

[14] Fiocco U., Sfriso P., Oliviero F. (2008). Co-stimulatory modulation in rheumatoid arthritis: the role of (CTLA4-Ig) abatacept. *Autoimmunity Reviews*.

[15] Arabshahi B., Silverman R. A., Jones O. Y., Rider L. G. (2012). Abatacept and sodium thiosulfate for treatment of recalcitrant juvenile dermatomyositis complicated by ulceration and calcinosis. *The Journal of Pediatrics*.

